# Secondary Sympatry as a Sorting Process

**DOI:** 10.1111/ele.70108

**Published:** 2025-04-11

**Authors:** Sean A. S. Anderson, Daniel R. Matute

**Affiliations:** ^1^ School of Biological Sciences Georgia Institute of Technology Atlanta Georgia USA; ^2^ Department of Biology University of North Carolina at Chapel Hill Chapel Hill North Carolina USA

**Keywords:** character displacement, coexistence, reinforcement, speciation, species sorting, sympatry

## Abstract

A much remarked‐upon pattern in nature is elevated trait disparity in sympatric relative to allopatric populations or species. Early explanations focused on secondary contact between allopatrically speciating taxa and emphasised adaptive divergence driven by costly interactions in sympatry (i.e., ‘character displacement’). Here we consider a related hypothesis, ‘species sorting’, which describes a bias in the outcome of secondary contact wherein lineages are unlikely to establish sympatry unless and until they evolve sufficient trait differences in allopatry. Sorting‐like processes are prevalent in community assembly theory but are more seldom discussed in the context of speciation and secondary sympatry. We first define ecological and reproductive species sorting as analogous to ecological and reproductive character displacement, and we synthesise ‘differential fusion’ and the ‘Templeton effect’ within this framework. Through the logic of coexistence and assembly theories, we distinguish the types of allopatry‐derived trait differences that will likely promote sympatry from those that likely will not, and we discuss biogeographic consequences of the latter. We then highlight new empirical approaches to distinguish sorting from displacement and survey the mixed evidence to‐date. We finally suggest key priorities for future research into the hypothesized role of species sorting as a generator of major biodiversity patterns.

## Graphical abstract

Early explanations for the frequent observation of exaggerated trait divergence in sympatric versus allopatric taxa focused on secondary contact between allopatrically speciating lineages and emphasised adaptive divergence driven by costly interactions (i.e., ‘character displacement’). Here we consider a related hypothesis, ‘species sorting’, wherein speciating lineages are unlikely to establish sympatry unless and until they evolve sufficient trait differences in allopatry. Our paper interrogates the logic behind the sorting hypotheses, synthesises various sorting‐related concepts as they pertain to speciation, highlights empirical approaches that can distinguish sorting from displacement, and finally evaluate the hypothesized role of sorting as a generator of major biodiversity patterns.

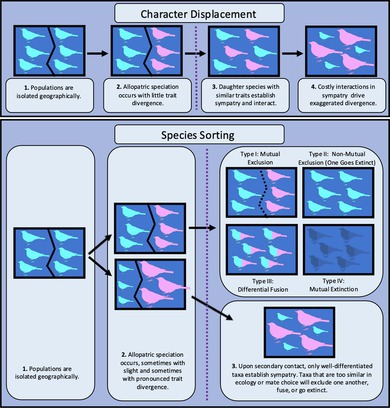

## 1 Introduction

Few patterns in nature have more stubbornly defied easy explanation than that of exaggerated divergence in the ecological or reproductive traits of closely related species in sympatry. Reports of the pattern are numerous and come in at least three versions. In the first, canonically described by Lack (1947) in Galapagos finches, allopatric populations of two species exhibit highly similar traits while sympatric populations show marked differences (Brown and Wilson 1956). In a second version, disparity in species‐mean trait values is greater on average for sympatric than for fully allopatric species pairs—a pattern seen, for example, in drosophilid premating barriers and across global datasets of avian functional traits (Coyne and Orr 1989, 1997; Matute and Cooper 2020; McEntee et al. 2018). The third form of the pattern is statistical overdispersion in the traits of species comprising an ecological guild (Holmes and Pitelka 1968; Strong et al. 1979; Dayan and Simberloff 1998, 2005). At least one version of the phenomenon is now recorded in dozens of species groups (Beans 2014; Brown and Wilson 1956; Dayan and Simberloff 2005; Pfennig and Pfennig 2012; Schluter 2000; Stuart and Losos 2013), and a testament to its generality is its taxonomic breadth: from monkeyflowers to monkeys and from barnacles to bats, observations of heightened trait differences in sympatry are, if not ubiquitous, certainly common enough to demand a theory. It is a pattern that begs explanation.

**FIGURE 1:**
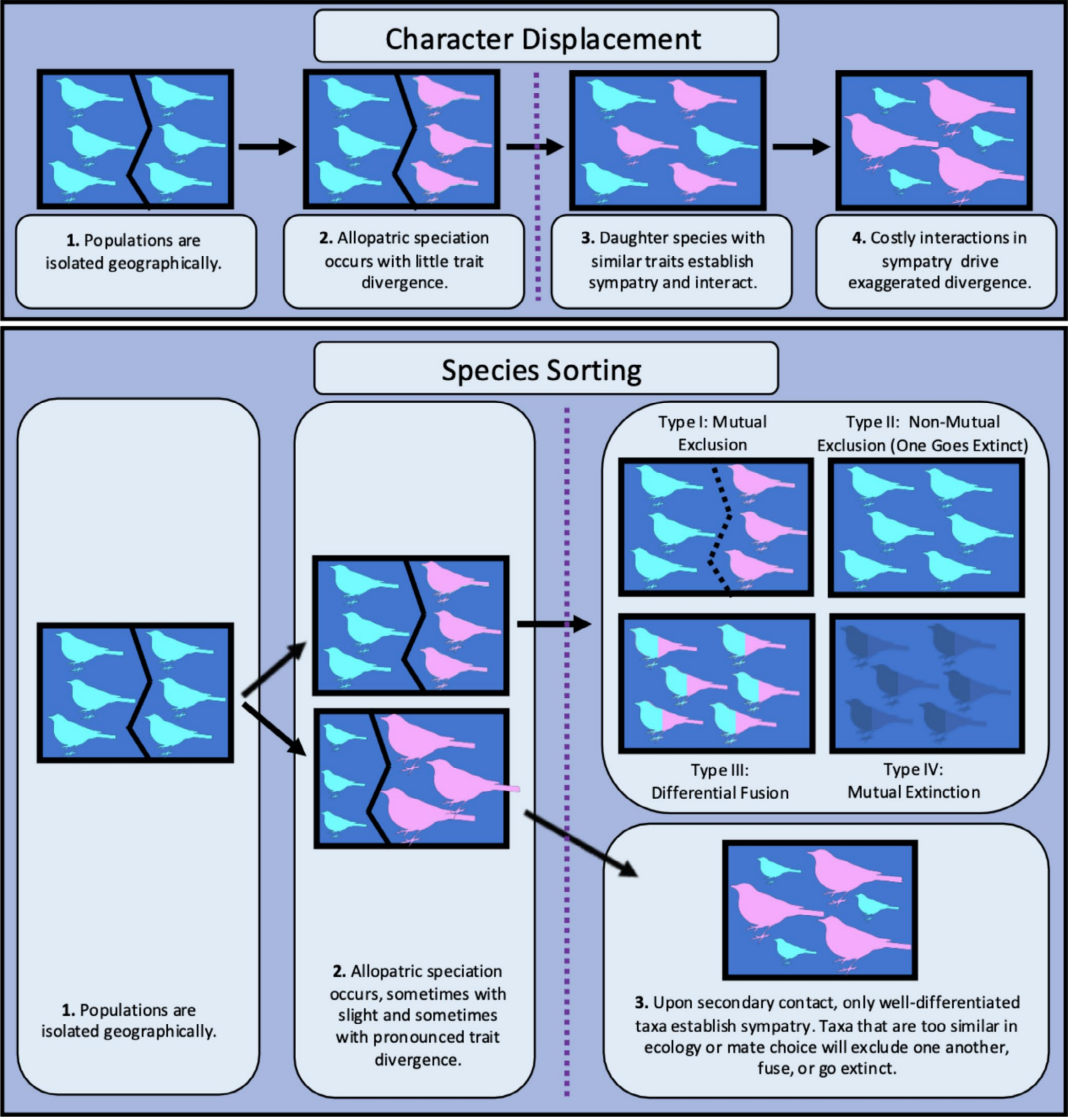
Conceptual distinction between sorting and displacement. Dark blue boxes are geographic ranges. Solid black lines within a range indicate a geographic boundary. Dotted black line indicates a boundary created by costly interactions between species (e.g., competition, reproductive interference). Purple dotted lines symbolise the timing of secondary geographic contact. Colour symbolises species identity for the diagram only and does not represent a biological attribute. Similarity in body size symbolises similarity in some critical aspect of ecology and/or reproduction. In both the displacement and the sorting scenarios, there are limits to similarity in sympatry. Under displacement, those limits drive the adaptive evolution of exaggerated differences in sympatric taxa; under sorting they prevent sympatry from being established. Species sorting predicts that taxa coming into secondary geographic contact when too similar tend to go extinct or face one of three other possible outcomes: (I) mutual exclusion, (II) the local extinction of one species due to competition or genetic swamping, (III) fusion into a single admixed lineage, or (IV) mutual extinction.

An answer was proposed by Brown and Wilson (1956) in the very first treatise on the pattern. Elevated divergence in sympatry, they suggested, “probably results most commonly from the first post‐isolation contact of two newly evolved cognate species. Upon meeting, the two populations interact…in such a way as to diverge further from one another where they occur together” (p.63). The pattern was thus framed as relating to secondary contact (following incipient or complete speciation in allopatry) and as resulting from divergent adaptation between interacting taxa (i.e., ‘character displacement’ sensu Grant 1972). The basic idea, which predates Brown and Wilson (1956), holds that taxa first establish sympatry while still similar in ecological or reproductive traits. As a consequence of their similarity, newly sympatric taxa compete for resources or interfere with one another reproductively, and the fitness^1^ costs imposed by these interactions drive the adaptive evolution of more pronounced trait differences (Figure 1). The displacement family of hypotheses (Box 1) has inspired volumes of work over the decades—‘ecological character displacement’ is a key component of adaptive radiation theory and a cornerstone of evolutionary ecology (Schluter 2000; Pfennig and Pfennig 2012), while ‘reinforcement’ has been hypothesized as a driver of late‐stage speciation since at least the Modern Synthesis (Fisher 1930, Dobzhansky 1937, Butlin 1987, Servedio and Noor 2003). But debate over the generality of character displacement continues (Stuart and Losos 2013), as alternative explanations are notoriously difficult to rule out.

BOX 1 **Terminology.**

(A) Definitions.

*Trait*: any characteristic relevant to coexistence, including observable phenotypes, physiological attributes, and characters like ‘mate choice’ or ‘foraging strategy’.

*Sympatry*: geographic co‐occurrence. Taxa whose ranges segregate in space (e.g., elevationally displacing mountain birds) do not co‐occur and so are not sympatric.

*Character Displacement*: adaptive evolutionary divergence between sympatric taxa in response to costly interspecific interactions caused by their similarity.

*Species Sorting*: a bias in the ability of species pairs to establish sympatry based on trait differences obtained in allopatry. Allopatric taxa that evolve sufficient trait differences tend to become sympatric on secondary contact while those lacking sufficient trait differences fail to because of (1) costly interspecific interactions caused by their similarity or (2) their tendency to fuse into a single taxon. This definition is common in speciation and evolutionary ecology, but community ecologists apply the same term different ways, such as describing environmental filtering (e.g., Leibold et al. 2004; Székely and Langenheder 2014; Wu et al. 2018).

*‘Ecological’ Species Sorting*: sorting in which ecological interactions between over‐similar taxa tend to preclude incipient sympatry; taxa with sufficient allopatrically derived ecological differences then become sympatric at a higher rate.

‘*Reproductive’ Species Sorting*: sorting in which reproductive interference between over‐similar taxa tends to preclude incipient sympatry; taxa with sufficient allopatrically derived reproductive differences then become sympatric at a higher rate.

*Reinforcement*: reproductive character displacement to avoid hybridisation.

*The Templeton Effect*: taxa with different degrees of pre‐ or postzygotic reproductive isolation differentially fuse or go extinct upon secondary contact (Templeton 1981).

*Differential Fusion*: a case of the Templeton effect in which the outcome of secondary contact is fusion (as opposed geographic exclusion or mutual extinction).

(B) The language of displacement: process or pattern?

It is useful to introduce species sorting in light of character displacement, but doing so means confronting the latter's troubled lexicon. Brown and Wilson (1956) originally defined character displacement as the pattern of exaggerated trait differences in the overlapping portion of two species' ranges. The authors hypothesized that a major cause of the pattern was adaptive divergence in sympatry due to costly interspecific interactions. Grant (1972) redefined character displacement as that very process of adaptive divergence (or convergence) in sympatry. Today, Grant's (1972) definition of character displacement is the most widely adopted, with the term referring to evolutionary divergence (not convergence) unless otherwise stated. The existence of two definitions can sow confusion: the process of character displacement defined by Grant (1972) is expected to generate the pattern of character displacement defined by Brown and Wilson (1956)—a pattern that has not been renamed since ‘character displacement’ was usurped.

Brown and Wilson (1956) also introduced the term ‘ecological displacement’ for the process of adaptive ecological divergence to avoid competition. That process is now termed ‘ecological character displacement’ (Slatkin 1980); its corollary for reproductive interactions is ‘reproductive character displacement’ (Crozier 1974; Pfennig and Pfennig 2012). ‘Reinforcement’ is character displacement for avoiding one reproductive interaction in particular—maladaptive hybridization—and its placement among these terms is debated; reinforcement is often considered reproductive character displacement but has also been defined as a distinct process (Butlin 1987; Butlin and Ritchie 1994). To cloud matters more, reinforcement is expected to create heightened premating isolation or exaggerated divergence in sexual signals in sympatry (Howard 1993), patterns that are sometimes called ‘reproductive character displacement’. This creates the unsatisfactory circumstance in which ‘ecological character displacement’ is a process but ‘reproductive character displacement’ at times refers to a pattern.

Despite perception to the contrary, there is no question about whether character displacement is a process or a pattern—it is explicitly defined as both in the literature, so the only option is to be clear about the definition one is using. We are sympathetic to Stuart et al.'s (2017) suggestion that Brown and Wilson's meaning be re‐adopted, but we fear the literature has gone so far in the other direction that doing so now would only bring further disorder to what is already a semantic state‐of‐anarchy. In this paper, we follow a terminology that captures the commonest usages in the literature: ‘character displacement’ is a catch‐all synonym for Grant's (1972) process of divergent character displacement, reproductive character displacement is a process, and reinforcement is a case of the latter.

One such alternative is that the initial establishment of sympatry itself tends to require marked trait differences, a concept behind the ‘species sorting’ family of hypotheses. ‘Species sorting’ is a bias in the ability of lineages to establish sympatry based on trait differences obtained in allopatry. It refers not to any single lineage pair but to the non‐random outcome of secondary contact across numerous pairs. Its premise is that upon secondary contact, over‐similar taxa tend to interfere with one another ecologically or reproductively and so fail to establish sympatry (Figure 1). Pairs that avoid interference by virtue of trait differences obtained in allopatry then become sympatric at a higher rate. Thus, in the sorting framework, the costs of similarity act as a collective sieve that ‘sorts’ lineage pairs into two classes: those that are sufficiently differentiated to stably coexist and those whose similarities prohibit coexistence. The former establish sympatry with a greater frequency than the latter. Elevated trait differences are in this case a prerequisite for sympatry rather than an evolutionary response to it, and species sorting is a pre‐adaption counterpart to character displacement (Box 2). Both processes can create all three patterns of elevated divergence in sympatry (Figure 1; Box 3) and posit limits to similarity. The key distinction is that under displacement, those limits drive divergence between taxa in sympatry, while under sorting, they tend to prevent taxa from becoming sympatric in the first place.

BOX 2 **Sorting or displacement, a thought experiment.**

Suppose we observe a sympatric lineage pair for which 50% of their marked trait differences resulted from divergence in allopatry, 50% from interactions in sympatry, and we knew that the allopatric divergence was essential for sympatry to be established. Would this represent sorting or displacement? The answer is both: this would be a case of species sorting *followed by* character displacement. The reason it is species sorting is that the establishment of sympatry is non‐random with respect to the trait: the question states that the allopatric trait divergence was essential for the establishment of sympatry (and implies that taxa lacking such trait differences did not become sympatric). Once the pair was sympatric, character displacement then accentuated the allopatrically derived trait differences. Thus, sorting was followed by displacement. Note that in practice, sorting cannot be determined empirically from a single instance of sympatry alone, a point on which we elaborate in the main text.

BOX 3 **Can sorting create Brown and Wilson's (1956) Pattern?**

It is well known that species sorting can generate overdispersion in the traits of sympatric guild members or elevated disparity in sympatric versus allopatric species pairs (versions 3 and 2 of the pattern described in the introduction). Less obvious is that sorting can generate Brown and Wilson's (1956) pattern of greater trait disparity between sympatric than between allopatric populations of the same two species. This pattern can indeed arise from sorting if at least one of the two species exhibits interpopulation trait variation prior to secondary contact. Populations in that case can be sorted such that only those interspecific populations that sufficiently differ in the relevant trait(s) are likely to become sympatric. Since Brown and Wilson's pattern can in principle result from sorting in this way, an important and classical criterion for demonstrating character displacement is to show that evolutionary shifts have actually occurred in sympatry (e.g., Schluter and McPhail 1992).

The concept of species sorting is not new (Box 4), but three factors support the need for a comprehensive review of the topic. The first is that none yet exists. Unlike the many wide‐ranging reviews of displacement (e.g., Butlin 1987; Dayan and Simberloff 2005; Germaine et al. 2018; Pfennig and Pfennig 2010, 2012; Schluter and McPhail 1992; Servedio and Noor 2003; Stuart and Losos 2013), the sorting hypothesis has not been thoroughly unpacked in both its ecological and reproductive versions. Several concepts that clearly fall under a sorting umbrella (‘ecological species sorting’, ‘differential fusion’, ‘the Templeton effect’) have yet to be synthesised, and the vocabulary is muddled. Second, species sorting is increasingly invoked to explain large‐scale patterns like trait divergence in evolutionary radiations (McEntee et al. 2018; Tobias et al. 2014), elevational turnover in montane birds (Reijenga et al. 2023), and rates of premating isolation in animals (Matute and Cooper 2021). The field can now benefit from a critical appraisal of these ideas and the evidence supporting them. Third, new statistical approaches promise to finally tease apart the signatures of sorting and displacement in comparative datasets (Anderson and Weir 2021, 2022; Reijenga et al. 2023). These advances have made species sorting not just an alternative explanation to be acknowledged but a falsifiable hypothesis in its own right.

We note that while species sorting is overshadowed by character displacement in the speciation literature, the same is not true for the literature of community ecology. The hypothesis that taxa are somehow sorted during community assembly is foundational in this context and underpins core concepts like the ‘habitat filter’ and newer ideas like community phylogenetics (Kraft et al. 2014, Cadotte and Tucker 2017, Cavender‐Bares et al. 2009). But this focus on filtering is only seldom applied to the problem of secondary contact in speciating taxa (i.e., ‘species sorting’ as defined here). This is perhaps unsurprising given the contrasting worldviews of community assembly and speciation research. Most assembly frameworks assume that speciation is complete. A set of fully realised species are assumed to exist in a regional pool from which local communities draw their members, and if those species pass through a filter, then that filter discriminates based on traits that are fixed (relative to the timescale of assembly). The taxa in question are not considered newly evolved and ‘cognate’, their contact is not assumed to be secondary (they may have encountered each other any number of times previously), and their ongoing evolution does not factor into the discussion. Speciation researchers, for their part, work from a foundational emphasis on divergent adaptation as a species generator (Darwin and Wallace 1858) and amid strong evidence of character displacement in some of the field's most comprehensively studied systems (Grant and Grant 2008; Schluter and McPhail 1992; Stroud et al. 2024). Community ecologists and speciationists have thus viewed the establishment of sympatry in distinct ways, and they have placed profoundly different emphases on the importance of sorting.

In this paper, we work to synthesise the two viewpoints by thinking in‐depth about species sorting during secondary contact following the allopatric stage of speciation. This is a context in which traits are not fixed and speciation is not necessarily complete, a context in which both displacement and sorting are possible. We consider what factors might dictate when one or both occur and how best to tell them apart with data. We also highlight similarities between displacement and sorting as well as the blurred line between them. Like most biological dichotomies, the sorting versus displacement distinction is not a strict binary (Box 2, 5). It is likely that the two are often complementary, with allopatrically derived trait differences fostering incipient sympatry and then being accentuated by character displacement (Lack 1947; Schluter 2000). Patterns of exaggerated trait differences in sympatry can then result from sorting among pairs, from displacement within pairs, or from a combination of the two. The processes are nonetheless unique and warrant separate consideration. In particular, whereas trait evolution is driven by costly interactions between the two taxa during character displacement, it is explicitly not under sorting, which acts on traits that have evolved by virtually any other means. It is worth considering how and when trait differences arising this way will determine coexistence.

BOX 4 **Origins of the species sorting hypotheses.**

A version of ecological species sorting was discussed at least as early as 1933 in a German‐language treatise on speciation by Bernhard Rensch (Rensch 1933; Mayr 1947; Pfennig and Pfennig 2012). Mayr, considering the niche partitioning observed in sympatric species flocks of several lakes, opined that “Rensch (1933) has indicated the right solution. It is that these species have come into contact only after … they had acquired their ecological differences” (Mayr 1947, 282).

The earliest discussion of reproductive species sorting is usually attributed to Templeton (1981). Evaluating the evidence for reinforcement, Templeton noted that its expected pattern of elevated premating isolation in sympatry could also result if taxa lacking that barrier go extinct or fuse upon secondary contact. It had long been appreciated that mechanisms of mate recognition could prevent extinction or fusion; A. R. Wallace, for instance, writes in *Darwinism* that “some means of easy recognition … enables the sexes to recognize their kind and thus avoid the evils of infertile crosses” (Wallace 1889 p.217). But it was Templeton who seems to have first realised the implied filtering effect. Templeton warned that when premating isolation was weak, “the most likely outcomes (extinction or fusion)” of secondary contact will “rapidly eliminate the appearance of a contact, thus leading to an observational bias for reinforcement” (Templeton 1981, 30). The process by which taxa with different degrees of reproductive isolation differentially fuse or go extinct upon secondary contact is a form of reproductive species sorting and is often called the ‘Templeton effect’ (Castillo 2017; Hollander et al. 2018; Nishimura et al. 2023).

BOX 5 **Character displacement via sorting of existing variation.**

A model of secondary sympatry that highlights the non‐exclusivity of character displacement and species sorting is that of displacement based on the sorting of preexisting intrapopulation trait variation (Rice and Pfennig 2007). In this model, secondary sympatry is established between taxa with partially overlapping trait distributions. Upon secondary contact, part of the trait distribution of one population is replaced by that of a population from a different species, where the first species is ecologically outcompeted or reproductively interfered‐with in that region of trait space (see Figure 2 of Rice and Pfennig 2007). Non‐overlapping sections of the original trait distributions remain intact. This scenario can be considered species sorting in that trait values required for coexistence evolved prior to the establishment of sympatry. However, trait evolution in sympatry has still occurred in at least one population in the form of a skewed reduction in trait variance, which causes a shift in the population mean—even in the absence of directional selection for more extreme trait values. Thus, the process is also unequivocally character displacement (Rice and Pfennig 2007).

## 2 The Cost of Similarity in Sympatry

The sorting and displacement families of hypotheses are both predicated on the idea that similarity breeds conflict by inciting negative interspecific interactions. The cost of those interactions manifests as reduced survival or reproduction of contact‐zone individuals that bear the similar traits. This cost can be shared more or less equally by the two taxa or can be borne disproportionately by a less dominant species.

‘Ecological’ and ‘reproductive’ character displacement are historically defined by the types of traits that diverge between interacting taxa, which is the same as saying the types of similarities that impose fitness costs. We define ‘ecological’ and ‘reproductive’ species sorting as caused by those same similarities (Box 1). Ecological similarities include overlapping resource requirements and impart fitness costs primarily through interspecific competition. This idea follows directly from the principles of limiting similarity (Macarthur and Levins 1967, Abrams 1983) and competitive exclusion (Hardin 1960). Similarity in reproductive traits like pollinator or mate‐choice preferences imparts fitness costs via reproductive interference (Cothran 2015; Gröning and Hochkirch 2008; Kyogoku 2015), of which hybridization is an extreme form. Hybridization is costly whenever hybrid genotypes have lower mean fitness than parental genotypes, but other forms of reproductive interference can be harmful. In animals, individuals can waste energy reserves or tempt predation when they advertise to heterospecifics, pursue inappropriate couplings, or unnecessarily defend nests and mating territory (Drury et al. 2015; Losin et al. 2016; Soni et al. 2024). And in plants, heterospecific pollen deposition can reduce reproductive output through several mechanisms (Runquist and Stanton 2013; Smith and Rausher 2008).

A question then arises, which is why the costs of similarity sometimes drive divergence between taxa in sympatry and other times prevent taxa from becoming sympatric altogether. The answer is that character displacement occurs conditionally (Liou and Price 1994). First, lineages must interact to generate divergent selection, so character displacement cannot occur if the costs of similarity are too great to allow even incipient sympatry (Milligan 1985, Figure 2). Second, from an evolutionary genetics perspective, character displacement is an increase in the relative fitness of genotypes encoding trait values that dampen negative interspecific interactions. This implies that (1) alternative genotypes must exist or arise via mutation in at least one taxon, (2) some of those genotypes must reduce the negative interaction, (3) populations must persist in sympatry long enough for selection to exert an evolutionary effect, and (4) displacing selection must not be diluted by gene flow from populations in allopatry (where alleles encoding similarity confer no disadvantage; see Pfennig and Pfennig 2012 for other criteria). When these conditions are unmet, reduced absolute fitness of available genotypes at the contact zone halts range expansion before sympatry is established. Secondary contact then becomes a sorting process. This conditional nature of character displacement can muddy the distinction between sorting and displacement by implying that capacity for the latter can be a basis for the former—that is, pairs can be sorted based on whether they will displace if necessary. Sorting of this kind would not (necessarily) be based on differences obtained in allopatry, so we do not focus on it further.

**FIGURE 2:**
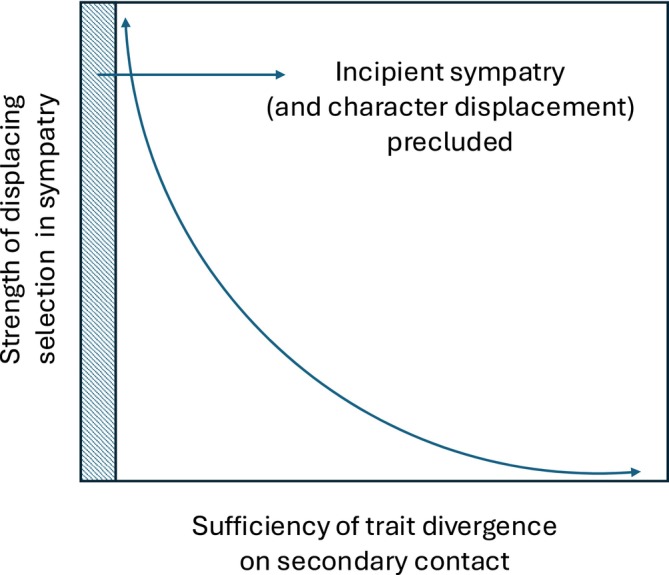
Complementarity of species sorting and character displacement. To the extent that limiting similarity exists, and some trait disparity is required for coexistence, then differences in a relevant trait can evolve prior to the establishment of sympatry, after the establishment of sympatry, or both. If trait differences are nearly sufficient for coexistence when secondary contact occurs, then taxa are under less pressure to displace. The strength of displacing selection should then increase as the sufficiency of allopatric trait differences declines—but only up to a point. If trait differences are wholly absent or ineffective on secondary contact, then incipient sympatry itself, and character displacement by extension, might often be precluded (shaded region). Note the x‐axis is ‘sufficiency’ of trait divergence, not magnitude, since greater divergence does not necessarily correspond to greater probability of coexistence on secondary contact (see ‘Avoiding the Cost of Similarity’).

There is an important exception to the rule that species sorting, like character displacement, implies a fitness cost to similarity. This exception is unique to a form of reproductive sorting called ‘differential fusion’ (Noor 1999). If taxa hybridise on secondary contact and lack sufficient postzygotic isolation (such that the mean fitness of hybrids is high or not prohibitively low), then the two lineages may simply merge (Liou and Price 1994). Character displacement cannot occur in such a case, but pairs can still be sorted by the process of differential fusion: taxa with sufficient mate choice differences will become sympatric while those that frequently hybridise will fuse upon secondary contact, thus resulting in the appearance of exaggerated mate‐choice differences in sympatry (Figure 1, ‘type III’). Note that while differential fusion requires no cost to similarity, it still implies a *limit* to similarity in that over‐similar taxa will often merge instead of coexisting as separate entities.

Limits to similarity are indeed fundamental to species sorting and character displacement; both hypotheses are sound only insofar as such limits exist. That there are real limits to reproductive similarity is uncontroversial. Taxa with too little reproductive isolation merge in sympatry (Seehausen 2006, Kearns et al. 2018), and weak premating barriers in postzygotically isolated taxa jeopardise coexistence (Liou and Price 1994; Servedio and Noor 2003; Irwin and Schluter 2022). Evidence for ecological limits to similarity is somewhat more ambiguous (Grime 2006, Wilson 2007) as clear signals are not always found in comparative datasets (Gotzenberger et al. 2012). Nonetheless, strong evidence for character displacement in some well‐studied systems (Galápagos finches, Grant and Grant 2008; sticklebacks, Schluter and McPhail 1992; spadefoot toads, Pfennig and Martin 2010; *Anolis*, Stroud et al. 2024) is *de facto* confirmation of a limit to similarity in those pairs, which raises an important point: neither character displacement nor species sorting claims that limiting similarity is necessarily pervasive. The hypotheses address only those cases in which a pattern consistent with limiting similarity (elevated divergence in sympatry) is observed, and they try to explain how the limit was avoided.

## 3 Avoiding the Cost of Similarity: Fortuitous Trait Divergence in Allopatry

Species sorting does not specify how traits diverge in allopatry. Trait evolution in the two lineages of a given pair can in principle be driven by any conceivable selective agent(s) *other* than interactions between the two taxa or even by drift. If there are limits to similarity, then sympatry on secondary contact is a matter of chance: trait differences that evolved for unrelated reasons must, by happenstance, temper or negate the interactions that would otherwise prohibit coexistence. But what kinds of trait differences will tend to do so? Verbal descriptions of species sorting (including our own) have focused on the magnitude of differentiation while giving less thought to the kinds of differences that foster sympatry. This is a mistake, as it is possible for substantial divergence in some traits to hinder rather than promote coexistence (Chesson 2000; Weber and Strauss 2016). In this section, we work to distinguish the types of allopatrically derived trait differences that likely promote sympatry from those that likely will not.

### 3.1 Allopatric Divergence in Ecological Traits

When assessing whether trait differences derived in allopatry will favour coexistence on secondary contact, two bodies of relevant theory are assembly theory and modern coexistence theory (MCT). Their concepts can be considered sequentially (Kraft et al. 2014). Assembly factors like abiotic habitat filters first determine if species end up in the same environment. Factors like ecological fitness and niche differences (Chesson 2000) then determine if the permeating taxa can stably coexist.

In the context of species sorting, assembly mechanisms dictate if secondary contact itself occurs. The coarsest filters through which taxa are drawn are abiotic, and here it is difficult to see how allopatric trait divergence can be favourable. Allopatric differentiation in abiotic niche axes like climate preferences can create substantially different habitat requirements and make taxa less likely to encounter one another in a given region—in effect, such taxa will not pass through the same habitat filters. If secondary contact were to occur in such a case, we would often expect the species to segregate along abiotic gradients rather than establish broad geographic overlap (Endler 1977).

Once taxa encounter one another, MCT predicts that whether trait differences favour coexistence depends on the extent to which they are ‘stabilising’ and ‘equalising’ (Chesson 2000; Adler et al. 2007). Trait differences are stabilising when they reduce inter‐ relative to intraspecific competition—only when population growth is more strongly limited by the latter can one lineage proliferate from low population size in the presence of the other (and vice versa), and the ability to do so is the operational definition of stable coexistence. Equalising differences are those that minimise ‘ecological fitness’ differences. ‘Fitness’ in MCT refers to the ratio of a lineage's per‐capita growth rate to its sensitivity to competition, where that sensitivity is the pace at which growth rate declines with declining resource abundance (Chesson 2000; Grainger et al. 2019). Fitness differences make coexistence less likely and can manifest in several ways, including disparity in foraging efficiency and parasite resistance. Allopatric divergence should typically be expected to increase fitness differences. Indeed, since ecological fitness differences start at zero (prior to the onset of speciation), allopatric divergence in any one trait can never be equalising relative to the starting state. However, trait differences arising on multiple axes can equalise each other. For example, if allopatric lineages diverge in both fecundity and competitive ability such that the weak competitor reproduces faster, then the second trait difference equalises the first (Chesson 2000).

**FIGURE 3:**
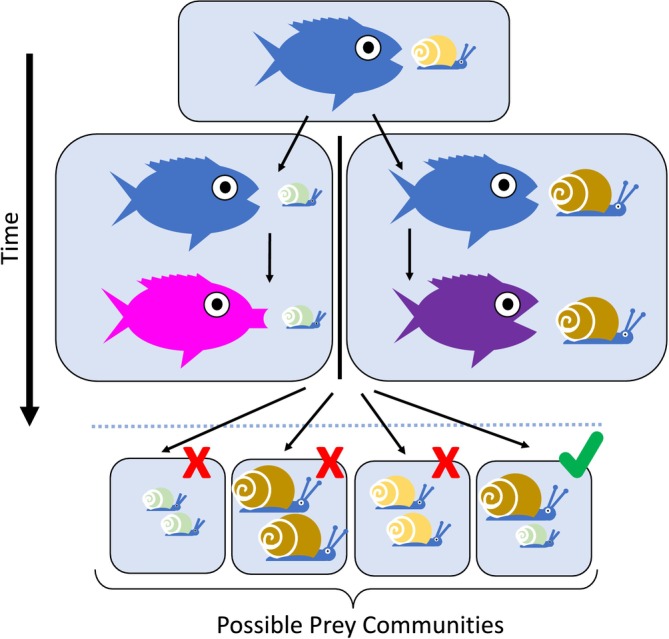
Coincidence of allopatric divergence with the environment of secondary contact. To promote secondary sympatry, allopatrically derived trait differences must be incidentally beneficial in the shared environment. As an illustration, we show a fish species adapted to preying on intermediate sized, yellow‐shelled snails. Populations of this species are subsequently isolated in allopatric environments containing distinct snail species: a small, green‐shelled snail or a large, brown‐shelled snail. Over time, as the lineages speciate, they adapt to their new prey items by evolving unique mouth parts. The dotted line indicates the timing of secondary contact. For these taxa to be eventually sorted into sympatry, they must meet in an environment containing both snail species (or prey species of similar size). Other prey communities will not support their coexistence. The community that supports coexistence is noted with a checkmark. The argument also holds for taxa diverging in reproductive traits like pollinator preference and can hold for continuous rather than discrete resource distributions.

Based on the factors considered here, we expect allopatric ecological differences to favour sympatry if they reduce competition without precluding taxa from readily overlapping and without favouring one lineage over another in a shared environment—however, chance is still a major factor. Specifically, trait differences obtained in allopatry must fortuitously match the conditions of the (secondarily) shared environment (Figure 3). One of those conditions is ecological opportunity. It would not be enough for speciating granivorous birds, for example, to diverge in seed size preference while in allopatry; seeds of roughly both sizes must also happen to be present and at sufficient density in the region of overlap for competition to be avoided. Too much allopatric divergence can then be just as costly as too little if taxa end up with resource requirements much more distinct than what is found and underexploited in the contact zone.

**FIGURE 4:**
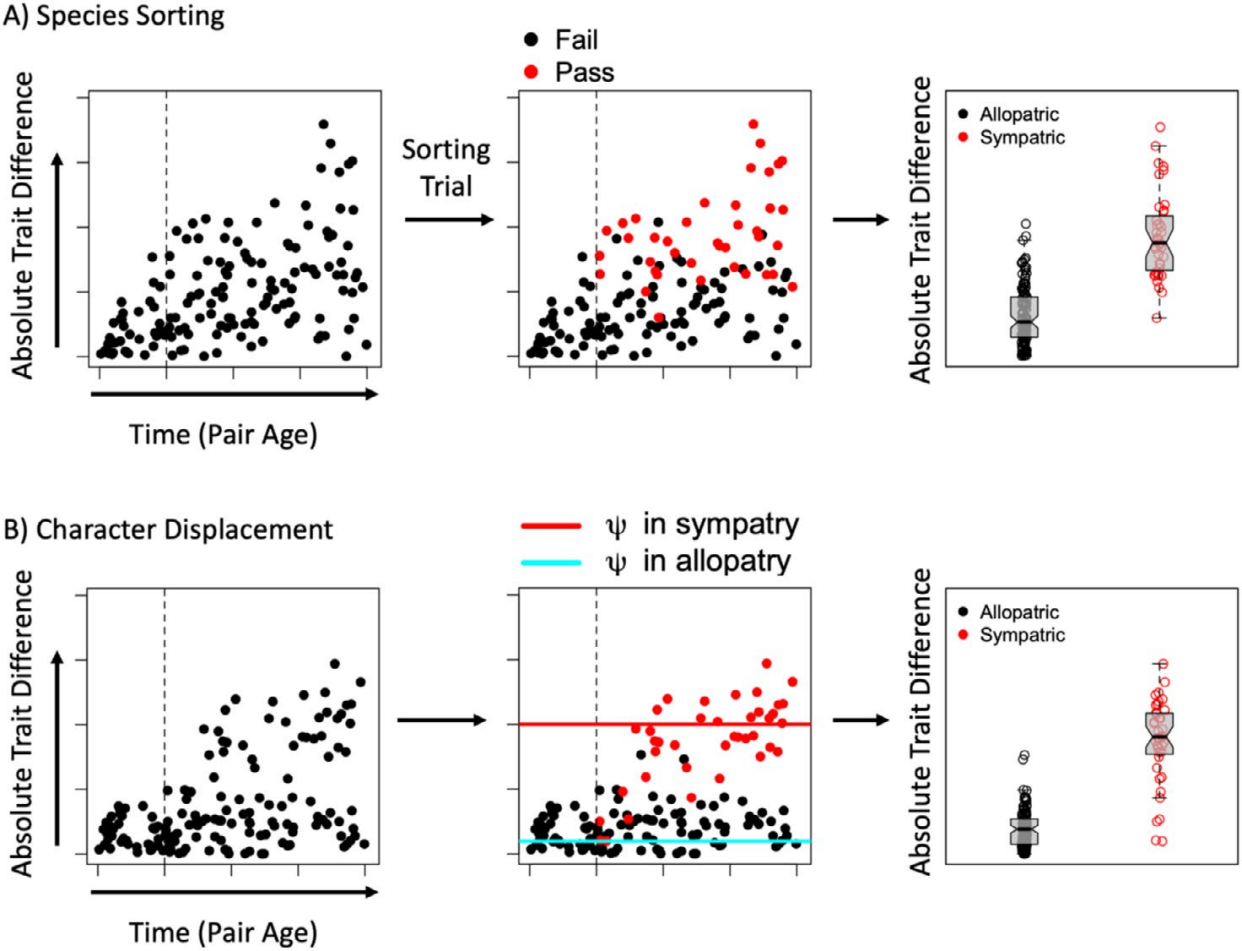
Generating expected trait differences under sorting and displacement. Each row is one run of a simulation procedure. Parameters were chosen for illustration purposes. Points are values of absolute trait differences for simulated sister pairs whose ages match those in the hypothetical dataset being analysed (for this illustration, they are drawn randomly). Dotted vertical lines denote the mean age of secondary contact, which can be estimated as a parameter. Simulations are repeated many times to generate expected distributions of trait disparity at each observed sister pair age. The fit of expected distributions to data is compared via likelihood or other model selection criteria. (A) Sorting is modelled as a Bernoulli trial in which success is the establishment of sympatry and the probability of success is a function of trait difference. To simulate differential fusion, one would remove taxa that failed the Bernoulli trial instead of resigning them to the allopatric group. (B) Character displacement is simulated as a change in the adaptive regime following the establishment of sympatry. Pairs are subject to stronger divergent selection once they become sympatric, where divergent selection is modelled as a non‐zero distance between the optima to which sisters in a pair adapt. The average distance between optima (ψ) in the two regimes is shown by the solid horizontal lines (cyan = allopatry; red = sympatry). Boxplots show that both procedures generate elevated trait differences in sympatry.

Thus, whether allopatrically derived ecological differences permit sympatry depends on at least four properties: (1) the extent of trait differentiation, (2) the niche axes on which differences evolved, (3) the relevance of those differences in the shared environment (Figure 2), and (4) the extent of divergence in ecological fitness. Our discussion has not considered coexistence frameworks in which niche differences are irrelevant (Bell 2000; Hubbell 2001) because their mechanisms are not expected to create elevated divergence in sympatry.

### 3.2 Allopatric Divergence in Reproductive Traits

Allopatric divergence favours the establishment of sympatry if it reduces reproductive interference. The types of trait divergence that do so depend on the life history of the organisms in question. In many animals, reproductive interference results from failures to distinguish con‐ and heterospecifics during mate choice (Gröning and Hochkirch 2008). Allopatric trait changes that increase mate recognition can then favour sympatry. Two avenues by which heightened recognition is achieved are increased sensitivity in mate discrimination and greater divergence in the sexual signals on which choosing is based (Price 2008; Grant and Grant 2008). Females of a speciating frog lineage, for example, might evolve to respond to a narrower range of courtship calls while in allopatry such that they ignore heterospecifics when contact resumes, or males might diverge in call frequency. Both changes might occur. In marine broadcast spawners, divergence in the mechanical structure of broadcasted gametes might be required to reduce reproductive interference (Palumbi 1994, 2008), whereas plants might diverge in pollinator preference or flower morphology. Allopatric divergence in microhabitat use (e.g., host‐plant divergence) or phenology can also lower reproductive interference and obviate the need for more acute mate recognition mechanisms.

Other forms of reproductive trait divergence can hinder the establishment of sympatry. This is especially true for traits in which divergence results in asymmetric reproductive interference. Such asymmetries not only depress the size of one population relative to the other but also handicap the ability of a lineage to increase when rare (Weber and Strauss 2016). Two insect lineages, for example, might allopatrically adapt to different degrees of sexual conflict such that a male–female arms race is waged in one lineage but not the other. Sexually aggressive males from the high‐conflict taxon could then ‘outcompete’ heterospecific males for access to females of both species when contact occurs. Low‐conflict females could then suffer the costs of producing unfit hybrids or of suboptimal mating frequency. Similarly, if the choosy sex in one animal lineage discriminates more strongly than that in the other, then the costs of reproductive interference could bear disproportionately on the weak discriminator and drive it to extinction.

A final consideration is whether reproductive differences obtained in allopatry can reduce interference within the conditions of the contact zone. For example, divergence in the male nuptial colours of two speciating fishes cannot reduce reproductive interference in water that is too murky. And if speciating plants diverge in pollinator preference while in allopatry, then both pollinator species must be present in the shared environment (and at reasonable population densities) for reproductive interference to be avoided. Sexual trait differences must also not impose exorbitant predation risk nor compromise the ability to obtain prey (Greather and Grey 1996). The mating signals of many plants and animals are tailored by sexual selection to be maximally detectable in a particular environment (Endler and Basolo 1998), thus the extent to which allopatric divergence in those signals translates into lowered reproductive interference depends in part on how acoustically, photically, and chemically similar the allopatric environments are to each other and to the contact zone.

### 3.3 Is Sorting More Plausible Than Displacement?

From a plausibility standpoint, it is unclear how commonly we should expect species sorting alone to explain patterns of elevated divergence in sympatry.

For ecological species sorting, sympatry would seem to hinge on an improbable Goldilocks scenario wherein the right amount of allopatric divergence happens to evolve on just the right niche axis and in the absence of notable divergence in ecological fitness. Sympatry thus relies on chance to (arguably) a much greater extent under ecological species sorting than under character displacement—sorting requires that trait evolution in allopatry is *incidentally* beneficial in the shared environment, but trait evolution under character displacement is directed by the environment itself. On the other hand, comparative analyses suggest that interspecific interactions commonly impose range limits in at least some species groups (Freeman et al. 2022, 2024; Pigot and Tobias 2013), suggesting that coexistence on secondary contact might indeed be rare and circumstantial. Secondary contact, moreover, is not necessarily a one‐off event requiring success on the first try. Taxa that do not immediately become sympatric might remain parapatric until one or both taxa hit on an evolutionary change that permits broader overlap, rather like trying a lock with different keys until one finally opens the door.

Reproductive species sorting is another story. There is a great range of ecological, phenotypic, and genetic differences that if obtained in allopatry might contribute to reproductive isolation or reduce reproductive interference on secondary contact. Some reproductive barriers are ‘intrinsic’ and effective regardless of the ecological theatre of the contact zone. Thus, the circumstances under which allopatrically derived trait differences permit coexistence would appear to be broader for reproductive than for ecological traits. Differential fusion in particular seems highly plausible and is perhaps a general phenomenon, though it is difficult to demonstrate, as the process erases evidence of contact and thus covers its own tracks.

Ultimately, how sorting or displacement contributes to elevated divergence in sympatry is an empirical problem. As we outline in a later section, it is a problem that is increasingly tractable.

## 4 Failure to Avoid the Cost of Similarity: Biogeographic Consequences of Species Sorting

Four outcomes are possible when taxa fail to establish sympatry during secondary contact. They are mutual exclusion, the local extinction of one taxon, the fusion of two lineages into one, and the extinction of both lineages (Figure 1).

Mutual exclusion occurs when two lineages meet geographically and neither cedes ground to the other (Figure 1, Type I). Potential reasons for the reciprocal halting of range expansion are many, and the rich literature on range limits is too vast to justly summarise here (Case and Taper 2000; Gaston 2009; Holt 2003; Louthan et al. 2015; Sexton et al. 2009), but a few scenarios are worth highlighting. First, mutual exclusion is expected when taxa allopatrically diverge in a niche axis like temperature or diet and then meet secondarily along a gradient in that character (Endler 1977). If each lineage is better adapted to its respective side of the contact zone, then neither gains a foothold in the other's range, and sympatry is prohibited. Second, there is growing evidence for ‘sexual exclusion’ (*sensu* Hochkirch et al. 2007) between taxa with postzygotic isolation and weak premating barriers (Mikkelsen and Irwin 2021, Pulido‐Santacruz et al. 2018). Maladaptive hybridization between such taxa renders the contact zone a demographic sink (Irwin 2020; Irwin and Schluter 2022, Liou and Price 1994; Goldberg and Lande 2007). Reinforcement could in principle solve this problem but has not done so in the cited examples. One reason might be genetic swamping—if the contact zone is narrow, then only a small proportion of either population is under selection for reduced hybridization, and Alleles bolstering mate discrimination might fail to establish (though evidence for swamping is mixed, Kottler et al. 2021).

A second outcome is local extinction of one lineage and its replacement by the other (type II sorting, Figure 1). Extinction due to ecological incompatibility is the expected result when destabilising ecological fitness differences outweigh stabilising niche differences; if taxa maintain similar resource requirements but allopatrically diverge in their ability to exploit those resources, then the weaker competitor will go locally extinct on secondary contact. Asymmetric reproductive interference can similarly exclude or drive a taxon to local extinction, a dynamic that has been explored experimentally in nematodes (Schalkowski et al. 2025) and has evidently been observed in a lake whitefish community (Frei et al. 2022); in a lake comprised of four species, one has recently gone extinct, but a substantial portion of its genome is now detected in all three surviving lineages. The negative frequency dependence of reproductive interference (Weber and Strauss 2016) implies that ecological and reproductive interactions might combine to knock‐out a species on secondary contact. If competition reduces the population of a weak competitor, then as the losing taxon becomes rarer, its members will have more opportunity for sexual interference with heterospecifics. The combined effects of ecological and reproductive interference can thus envelop a poor competitor in a feedback loop that terminates at local extinction.

A third outcome of insufficient differentiation in allopatry is the simple fusing of two lineages into one (type III sorting, Figure 1). Fusion occurs when two taxa hybridise in the absence of robust postzygotic reproductive isolation and thus pay no prohibitive fitness cost. The genomic contribution made by each parent to the resulting hybrid population varies along a continuum. At one end of the spectrum, the hybrid lineage bears a roughly equal mosaic of the parental genomes, as has been observed following mass hybridization in swordtail fishes (Schumer et al. 2016), North American ravens (Kearns et al. 2018), and stickleback ecotypes (Gow et al. 2006; Taylor et al. 2006). At the opposite end, the genome of one parent dominates or completely swamps that of the other, as has been experimentally observed in some *Drosophil*a (Matute et al. 2020). From the genomic perspective, the latter case is equivalent to the local extinction of one lineage (Mallet 2005; Rhymer and Simberloff 1996; Todesco et al. 2016). How to predict where along this spectrum a fused lineage will fall is not yet clear. An important question is indeed how the demographic, ecological, and genomic attributes of the parents affect the ecological and genomic attributes of the resulting hybrid lineage.

The last possibility is that costly interactions drive both lineages extinct upon secondary contact. Mutual extinction would occur when over‐similar taxa neither segregate geographically nor undergo character displacement. Absolute fitness in both populations is then reduced such that each declines terminally. Presumably, when population growth is strongly and mutually limited by negative interspecific interactions, then the extinction of one lineage will trigger the proliferation of the other. We might then expect mutual extinction to occur under limited circumstances, for example when both taxa are driven to low population sizes and then each goes extinct due to stochastic population fluctuations or Allee effects. Alternatively, widespread hybridization among certain taxa could cause the populations to merge into a swarm with low mean fitness that ultimately falls to extinction.

## 5 Empirical Approaches to Test for Species Sorting

Researchers attempting to empirically distinguish species sorting from character displacement are faced with two immediate challenges. First, both processes are expected to result in a pattern of elevated trait divergence in sympatry, and second, pairs that are sorted into sympatry can then undergo character displacement. The similarity in the predictions of the two hypotheses has long frustrated empiricists and partly explains why strong support for character displacement is limited to surprisingly few cases despite intense interest in that topic (Stuart and Losos 2013). But the ambition is not futile. New statistical methods have begun to highlight the subtle but distinct signatures generated by sorting and displacement. Given appropriate datasets, researchers can now test alternative hypotheses regarding the prevalence of the two processes in nature.

Tests of species sorting require a collection of both allopatric and sympatric pairs of populations or species. To understand why, imagine a dataset that contained just one sympatric pair. Even if we knew this pair's trait differences evolved in allopatry, we could not infer a statistical bias in the outcome of secondary contact from this observation alone; we would have zero usable degrees of freedom. Species sorting is a filtering process and so is only apparent in the collective. Unlike character displacement—for which empirical tests technically require just one sympatric population (Schluter and McPhail 1992; Howard 1993; Pfennig and Pfennig 2012; Hopkins 2013) – empirical tests of sorting require comparisons among multiple instances of both sympatry and allopatry.

In this comparative context, support for species sorting is often inferred when the predictions of character displacement are not borne out. For example, reinforcement predicts that prezygotic but not postzygotic barriers will be stronger in sympatry (Coyne and Orr 1989, 1997; see Coyne 1974 for a possible exception). It further predicts that when the cost of hybridization is asymmetric, then a concordant asymmetry will emerge such that the taxon paying the greater cost will also exhibit greater mate discrimination (Yukilevich 2012). The observation that postzygotic isolation is also stronger or that no concordant asymmetry arises can then suggest that species sorting has played a role (Matute and Cooper 2021).

A more direct approach is to generate datasets of absolute trait differences for numerous allopatric and sympatric sister pairs and compare the fit of the data to the predictions of sorting and displacement (Anderson and Weir 2021, 2022). The datasets must contain pairs of various ages for those predictions to be distinguished. The predictions of species sorting are generated by simulation (Figure 4). Trait divergence is first simulated many times for pairs at each age found in the dataset to create background expectations for sisters that have not come into contact. Secondary contact is then modelled as a Bernoulli trial in which the probability of ‘success’ (establishment of sympatry) increases with trait disparity. Successful and unsuccessful pairs are finally gathered to form sympatric and post‐contact allopatric distributions of trait divergence, respectively (empirical trait differences for allopatric pairs are fit to both the pre‐ and post‐contact allopatric distributions generated by simulation; the resulting likelihoods are weighted by the probability that a pair has come into secondary contact, which is an increasing function of age). The predictions of character displacement are obtained numerically or through simulation of a model in which all pairs begin as allopatric and some then transition to sympatry and a regime of stronger divergent selection (Figure 4). Wait‐times to this shift are drawn from a Weibull or similar distribution whose shape can be informed by empirical estimates of times to sympatry (e.g., Weir and Price 2011). The predictions of the two hypotheses are in some ways very similar (Figure 4); nonetheless, sorting and displacement predict distinct patterns in the distributions of expected trait disparity over time (see Figure 2 in Anderson and Weir 2021), and the fit of these expectations to data serves as the basis for model comparison.

A separate empirical approach involves assigning discrete states to numerous sister pairs of various ages based on their geography and ecological similarity (Reijenga et al. 2023). For example, pairs that are allopatric and ecologically similar are assigned “State 1”. Other states include allopatric and ecologically distinct (“State 3”) and sympatric and ecologically distinct (“State 4”). Datasets are then analysed with continuous‐time Markov models in which pairs transition along a succession of states, beginning in allopatry and ending in sympatry. The journey from the first to the final state involves passing through intermediate states, and different processes predict different pathways. For instance, species sorting predicts a transition from State 1 to State 3 to State 4; character displacement predicts a different route that includes no passage through State 3. A promising feature of this framework is that it can yield estimates of the relative contribution of each process based on how the prevalence of different states varies with pair age.

Other tests of species sorting leverage the tools of phylogenetics. A classic early example is Losos' (1990, 1992) work in Lesser‐Antillean anoles. Many islands of the Lesser Antilles contain either one intermediate‐sized anole or two anoles with pronounced size disparity. Evidence for sorting and displacement can be compared by constructing a phylogeny of the species in the region. If size divergence occurred in situ on every island, as expected from displacement, then many lineages should be more closely related to their sympatric partner than to other taxa of their size class. But if the smaller species all belong to one clade and the larger species to another, then the simplest explanation is that size divergence occurred once and islands were colonised by sorting among the descendants (this was indeed the result, which was also reported in New Guinean kingfishers (Linck et al. 2020); Losos (1992) also estimated (with low confidence) that ancestral size divergence coincided with the transition from one‐ to two‐species islands, suggesting that character displacement between anole ancestors might have created the very trait differences by which descendants were subsequently sorted). A second, more modern approach takes advantage of phylogenetic models of macroevolutionary ‘bursts’ in trait values (e.g., Uyeda et al. 2011; Brennan et al. 2024). The timing of evolutionary bursts can be compared to estimates of times‐to‐sympatry. Species sorting predicts that bursts of trait evolution will predate the establishment of sympatry; character displacement predicts the opposite (McEntee et al. 2018).

Several approaches have been put forth to assess the role of interspecific interactions in structuring assembly more broadly (i.e., not just during speciation). The 1970's and 80's saw an explosion of new methods for inferring competition as an important force in community assembly, along with an infamous debate over their use, all of which is reviewed elsewhere (Dayan and Simberloff 2005, Gotelli and Graves 1996, Lewin 1983, Losos et al. 1989, Stone and Roberts 1990, Strong et al. 1979, Weiher and Keddy 1995). A common theme was to test for even spacing in the resource‐exploitation traits of species in an ecological guild, the logic being that competition will prevent species with tightly clustered traits (and, by assumption, resource requirements) from co‐occurring. More‐recent approaches based on similar logic include tests of community phylogenetic dispersion (Cavender‐Bares et al. 2009; Webb et al. 2002), which is subject to its own debate (Gerhold et al. 2015; Mayfield and Levine 2010), and trait‐based community assembly (Borges et al. 2019, Forsyth and Gilbert 2021, Funk et al. 2017). Most methods infer a limit to similarity but are not designed to distinguish sorting from displacement. The work of Case and Sidell (1983) is an exception that clearly articulates the expectation for the two processes (“size adjustment” versus “size assortment”) in the context of archipelagos. Their approach entails measuring trait distributions for each island's population for each species in a guild and then bootstrapping to generate null expectations of ‘no species sorting’ and ‘no character displacement’ against which observed trait differences are compared.

## 6 What Is the Evidence for Species Sorting?

The empirical study of species sorting, as we have defined it here, is in its infancy. Deliberate tests of sorting that consider the possibility of character displacement and other mechanisms were largely impossible until only recently. As a consequence, they have been conducted in only a few studies to date and in only a few taxonomic groups, with birds being overrepresented. The results are mixed and somewhat method‐dependent. Our hope is that by highlighting this current lack of evidence and by pointing out several new methods, we can help spur researchers to pursue more empirical tests of the sorting hypothesis.

There is yet little indication from model‐based studies that species sorting is a prevalent mechanism driving patterns of exaggerated ecological divergence in sympatry. In a comparative analysis of bill shape divergence in New World birds, support for character displacement strongly outweighed that for species sorting (Anderson and Weir 2021), indicating that character displacement was the dominant but not necessarily sole process responsible for patterns of divergence. Results from Reijenga et al. (2023) based on elevational turnover in montane birds echoed this interpretation. Using the Markov‐model framework described previously, the authors estimated that 18% of pairs were best described by the transitional pathway predicted by species sorting while 81% were better described by the pathway predicted by character displacement alone. While species sorting did not come out as the major determinant, the 18% estimate indicates a substantial contribution of species sorting to a major biodiversity pattern and suggests that its effects are both detectable and non‐trivial. By contrast, in a much wider empirical analysis of numerous traits across allopatric pairs of terrestrial vertebrates, virtually no support was detected for the expected statistical signature of species sorting (Anderson and Weir 2022).

Other, less‐direct types of analyses have reported results consistent with a sorting process. In comparative speciation datasets of *Drosophila*, *Lepidoptera*, and *Bufo*, empirical estimates of faster rates of postzygotic isolation in sympatry are not expected by reinforcement and suggest instead the action of differential fusion, perhaps in concert with other processes (Matute and Cooper 2021). In the radiation of neotropical ovenbirds, the observed difference in trait disparity between sympatric and allopatric taxa disappeared after controlling for evolutionary age, a result interpreted as evidence for sympatry being established sometime after (and perhaps because of) the gradual accumulation of trait differences in allopatry (Tobias et al. 2014). In birds more generally, plumage dichromatism between sister species predicts the rate at which narrow geographic range overlap, but not broad sympatry, is established (Cooney et al. 2017). Such a pattern was interpreted as reflecting differential fusion wherein plumage colour differences are sufficient to prevent taxa from fusing upon contact but are not themselves sufficient to permit widespread coexistence, which might require ecological differences.

Even in favourable cases, however, it is often difficult to rule out alternative explanations. In an impressive analysis of global avian diversity, McEntee et al. (2018) found that body mass divergence in sister species occurs not gradually but in pulses that were inferred to mostly precede the establishment of sympatry, a pattern predicted by sorting. The wait times to an evolutionary pulse were estimated from a model and compared to the proportion of sister pairs that are sympatric at a given age; the authors show that by the time a pulse is likely to have occurred, only a small proportion of sister pairs have typically become sympatric. This finding in a major radiation represents the broadest support to date for any sorting process, but two issues leave this result open to interpretation. First, wait times in the model were drawn from an exponential distribution, which biases those times toward zero. The exponential is widely used to model wait times to events in macroevolutionary studies, but it dictates that shorter wait times are exponentially more frequent than longer ones—in the context of the pulse model, the time step with the greatest probability of observing an evolutionary pulse is indeed the first time step. Second, the authors note that large‐magnitude pulses of trait evolution are expected only for a small proportion of pairs in their model. Thus, even if wait time estimates were unbiased, the small proportion of pairs in which large pulses have occurred might be the same small proportion that are already sympatric. More work is needed to reject these possibilities. A separate study in birds shows that differential fusion likely accounts for the apparent rapid establishment of sympatry for pairs at high latitudes, but the authors note their data cannot rule out displacement (Martin et al. 2010). Similarly, avian species pairs without migratory divides exhibit greater trait differences, a pattern consistent with differential fusion wherein taxa lacking migratory divides are more likely to hybridise and therefore to fuse when such differences are absent (Delmore et al. 2015). Here again, sorting is plausible, but character displacement has not been ruled out.

## 7 Conclusions and Research Priorities

Like many problems in ecology and evolution, our understanding of secondary sympatry is probably influenced by a strong survivorship bias. Just as our interpretations of the fossil record, the shapes of phylogenies, and the allele frequencies of populations are affected by the many species and forms and mutations that arose and vanished without leaving a trace, so too is our interpretation of secondary sympatry affected by the countless attempts at coexistence that likely got no farther than secondary contact or that ended in lineage fusion. If there is a non‐random average difference between the pairs that successfully established sympatry and those that did not, then a filtering process has occurred. Our aim in this paper was to discuss in some depth this particular filtering process, which we refer to as species sorting.

Species sorting and character displacement both generate patterns of exaggerated divergence in sympatric taxa, and the two mechanisms occur for the same reason: lineages that are too similar cannot coexist indefinitely. The two processes are also not mutually exclusive. But despite their similarities, sorting and displacement differ notably in their implications for processes like trait divergence and the establishment of sympatry among close relatives. Sympatric coexistence, in turn, can be a rate‐limiting step in the diversification cycle, as it allows continued range expansion and the opportunity for future rounds of allopatric speciation to occur (Price 2008; Weir and Price 2011). Sorting and displacement can thus be said to differ in their implications for the factors affecting the build‐up of biodiversity, and we suggest it is worth the effort to more broadly measure their relative impacts.

An obvious research priority is the further development of methods to distinguish sorting from displacement and the application of these tests to a much broader set of taxonomic systems. Existing statistical tests of sorting require estimates of divergence times between sisters and, therefore, calibrated molecular clocks and phylogenetic hypotheses. These requirements have been met in birds for some time, but many more systems are now sufficiently characterised such that tests of sorting can proceed. There is a dearth of tests in plants in particular, and we hope to see this change in the near future. A great priority will be to devise tests of differential fusion, a mechanism that we suspect is near‐ubiquitous but that is difficult to analyse and is therefore the least well‐studied of any sorting process. Improvements and elaborations on the existing models of sorting are also needed and represent an opportunity for fast progress. A point that we hope readers take home is that species sorting is no longer a back‐up explanation to be duly acknowledged in the discussion section of papers on displacement; it is now a testable hypothesis in its own right.

In the context of speciation, species sorting has lived in the shadow of character displacement. It has been discussed in less depth in the literature and is much less frequently tested. Until recently, evidence for sorting, such as it was, came mostly from the failure of empirical tests to meet all the criteria for displacement. This is starting to change. Studies designed to deliberately test for species sorting and assess its impact relative to displacement are slowly on the rise. While there is little evidence for sorting as a major contributor to the exaggerated divergence observed in sympatric taxa, there is now at least some indication that it has a detectable and non‐trivial effect on this pattern. We suspect it is likely that with additional work, sorting processes will come to share at least some space in the spotlight with their displacement‐based counterparts. After a prolonged period of empirical isolation, there is room for coexistence among these conceptual close relatives.

## Author Contributions

S.A.S.A. and D.R.M. conceived of the synthesis. S.A.S.A. wrote the paper with input from D.R.M.

### Peer Review

The peer review history for this article is available at https://www.webofscience.com/api/gateway/wos/peer‐review/10.1111/ele.70108.

## Data Availability

The authors have nothing to report.

## Endnotes

^1^
Unless otherwise stated, we use ‘fitness’ in the evolutionary genetics sense of the word (Orr 2009); we specify ‘ecological fitness’ for fitness from coexistence theory (Chesson 2000).

## References

[ele70108-bib-0001] Abrams, P. 1983. “The Theory of Limiting Similarity.” Annual Review of Ecology and Systematics 14: 359–376.

[ele70108-bib-0002] Adler, P. B. , J. HilleRisLambers , and J. M. Levine . 2007. “A Niche for Neutrality.” Ecology Letters 10: 95–104. 17257097 10.1111/j.1461-0248.2006.00996.x

[ele70108-bib-0003] Anderson, S. A. S. , and J. T. Weir . 2021. “Character Displacement Drives Trait Divergence in a Continental Fauna.” Proceedings of the National Academy of Sciences of the United States of America 118: e2021209118. 33963076 10.1073/pnas.2021209118 PMC8157990

[ele70108-bib-0004] Anderson, S. A. S. , and J. T. Weir . 2022. “The Role of Divergent Ecological Adaptation During Allopatric Speciation in Vertebrates.” Science 378: 1214–1218. 36520892 10.1126/science.abo7719

[ele70108-bib-0006] Beans, C. M. 2014. “The Case for Character Displacement in Plants.” Ecology and Evolution 4: 862–875. 10.1002/ece3.978 PMC3967910 24683467

[ele70108-bib-0007] Bell, G. 2000. “The Distribution of Abundance in Neutral Communities.” American Naturalist 155: 606–617. 10.1086/303345 10777433

[ele70108-bib-0008] Borges, I. L. , L. Z. Forsyth , D. Start , and B. Gilbert . 2019. “Abiotic Heterogeneity Underlies Trait‐Based Competition and Assembly.” Journal of Ecology 107: 747–756.

[ele70108-bib-0009] Brennan, I. G. , D. G. Chapple , J. S. Keogh , and S. Donnellan . 2024. “Evolutionary Bursts Drive Morphological Novelty in the World's Largest Skinks.” Current Biology 34: 1–12. 39137786 10.1016/j.cub.2024.07.039

[ele70108-bib-0010] Brown, W. L. , and E. O. Wilson . 1956. “Character Displacement.” Systematic Zoology 5: 49–64.

[ele70108-bib-0011] Butlin, R. 1987. “Speciation by Reinforcement.” Trends in Ecology & Evolution 2: 8–13. 21227808 10.1016/0169-5347(87)90193-5

[ele70108-bib-0012] Butlin, R. K. , and M. G. Ritchie . 1994. “Behaviour and Evolution.” In Behaviour and Speciation, edited by P. J. B. Slater and T. R. Halliday , 43–79. Cambridge University Press.

[ele70108-bib-0013] Cadotte, M. W. , and C. M. Tucker . 2017. “Should Environmental Filtering Be Abandoned?” Trends in Ecology & Evolution 32: 429–437. 28363350 10.1016/j.tree.2017.03.004

[ele70108-bib-0014] Case, T. J. , and R. Sidell . 1983. “Pattern and Hance in the Structure of Model and Natural Communities.” Evolution 37: 832–849. 28568130 10.1111/j.1558-5646.1983.tb05604.x

[ele70108-bib-0015] Case, T. J. , and M. L. Taper . 2000. “Interspecific Competition, Environmental Gradients, Gene Flow, and the Coevolution of Species' Borders.” American Naturalist 155: 583–605. 10.1086/303351 10777432

[ele70108-bib-0016] Castillo, D. M. 2017. “Factors Contributing to the Accumulation of Reproductive Isolation: A Mixed Model Approach.” Ecology and Evolution 7: 5808–5820. 28811884 10.1002/ece3.3093 PMC5552923

[ele70108-bib-0017] Cavender‐Bares, J. , K. H. Kozak , P. V. Fine , and S. W. Kembel . 2009. “The Merging of Community Ecology and Phylogenetic Biology.” Ecology Letters 12, no. 7: 693–715. 10.1111/j.1461-0248.2009.01314.x. 19473217

[ele70108-bib-0018] Chesson, P. 2000. “Mechanisms of Maintenance of Species Diversity.” Annual Review of Ecology and Systematics 31: 343–366.

[ele70108-bib-0019] Cooney, C. R. , J. A. Tobias , J. T. Weir , C. A. Botero , and N. Seddon . 2017. “Sexual Selection, Speciation and Constraints on Geographic Range Overlap in Birds.” Ecology Letters 20: 863–871. 28513066 10.1111/ele.12780

[ele70108-bib-0020] Cothran, R. D. 2015. “The Importance of Reproductive Interference in Ecology and Evolution: From Organisms to Communities.” Population Ecology 57: 339–341.

[ele70108-bib-0021] Coyne, J. A. 1974. “The Evolutionary Origin of Hybrid Inviability.” Evolution 28: 505–506. 28564848 10.1111/j.1558-5646.1974.tb00781.x

[ele70108-bib-0022] Coyne, J. A. , and H. A. Orr . 1989. “Patterns of Speciation in Drosophila.” Evolution 43, no. 2: 362–381. 10.1111/j.1558-5646.1989.tb04233.x. 28568554

[ele70108-bib-0023] Coyne, J. A. , and H. A. Orr . 1997. “Patterns of Speciation in Drosophila Revisited.” Evolution 51: 295–303. 28568795 10.1111/j.1558-5646.1997.tb02412.x

[ele70108-bib-0024] Crozier, R. H. 1974. “Niche Shape and Genetical Aspects of Character Displacement.” American Zoologist 14: 1151–1157.

[ele70108-bib-0025] Darwin, C. R. , and A. R. Wallace . 1858. “On the Tendency of Species to Form Varieties; and on the Perpetuation of Varieties and Species by Natural Means of Selection.” Journal of the proceedings of the Linnean Society. Zoology 3: 45–62.

[ele70108-bib-0026] Dayan, T. , and D. Simberloff . 1998. “Size Patterns Among Competitors: Ecological Character Displacement and Character Release in Mammals, With Special Reference to Island Populations.” Mammal Review 28: 99–124.

[ele70108-bib-0027] Dayan, T. , and D. Simberloff . 2005. “Ecological and Community‐Wide Character Displacement: The Next Generation.” Ecology Letters 8: 875–894.

[ele70108-bib-0134] Delmore, K. E. , H. L. Kenyon , R. R. Germain , and D. E. Irwin . 2015. “Phenotypic Divergence During Speciation is Inversely Associated With Differences in Seasonal Migration.” Proceedings of the Royal Society B 282: 20151921. 26559951 10.1098/rspb.2015.1921 PMC4685813

[ele70108-bib-0028] Dobzhansky, T. 1937. Genetics and the Origin of Species. Columbia University Press.

[ele70108-bib-0029] Drury, J. P. , K. W. Okamoto , C. N. Anderson , and G. F. Grether . 2015. “Reproductive Interference Explains Persistence of Aggression Between Species.” Proceedings of the Royal Society B 282: 20142256. 25740887 10.1098/rspb.2014.2256 PMC4375855

[ele70108-bib-0030] Endler, J. A. 1977. Geographic Variation, Speciation, and Clines. Princeton University Press. 409931

[ele70108-bib-0031] Endler, J. A. , and A. L. Basolo . 1998. “Sensory Ecology, Receiver Biases and Sexual Selection.” Trends in Ecology & Evolution 13: 415–420. 21238370 10.1016/s0169-5347(98)01471-2

[ele70108-bib-0032] Fisher, R. A. 1930. The Genetical Theory of Natural Selection. Clarendon Press.

[ele70108-bib-0033] Forsyth, L. Z. , and B. Gilbert . 2021. “Parallel Responses of Species Diversity and Functional Diversity to Changes in Patch Size Are Driven by Distinct Processes.” Journal of Ecology 109: 793–805.

[ele70108-bib-0034] Freeman, B. G. , E. T. Miller , and M. Strimas‐Mackey . 2024. “Interspecific Competition Shapes Bird Species' Distributions Along Tropical Precipitation Gradients.” Ecology Letters 27: e14487. 39086139 10.1111/ele.14487

[ele70108-bib-0035] Freeman, B. G. , M. Strimas‐Mackey , and E. T. Miller . 2022. “Interspecific Competiton Limits Bird Species' Ranges in Tropical Mountains.” Science 377: 416–420. 35862538 10.1126/science.abl7242

[ele70108-bib-0036] Frei, D. , R. De‐Kayne , O. M. Selz , O. Seehausen , and P. G. D. Feulner . 2022. “Genomic Variation From an Extinct Species Is Retained in the Extant Radiation Following Speciation Reversal.” Nature Ecology & Evolution 6: 461–468. 35210577 10.1038/s41559-022-01665-7

[ele70108-bib-0037] Funk, J. L. , J. E. Larson , G. M. Ames , et al. 2017. “Revisiting the Holy Grail: Using Plant Functional Traits to Understand Ecological Processes.” Biological Reviews 92: 1156–1173. 27103505 10.1111/brv.12275

[ele70108-bib-0038] Gaston, K. J. 2009. “Geographic Range Limits: Achieving Synthesis.” Proceedings of the Royal Society B 276: 1395–1406. 19324809 10.1098/rspb.2008.1480 PMC2677218

[ele70108-bib-0039] Gerhold, P. , J. F. Cahill Jr , M. Winter , I. V. Bartish , and A. Prinzing . 2015. “Phylogenetic Patterns Are Not Proxies of Community Assembly Mechanisms (They Are Far Better).” Functional Ecology 29: 600–614.

[ele70108-bib-0040] Germaine, r. M. , J. L. Williams , D. Schluter , and A. L. Angert . 2018. “Moving Character Displacement Beyond Characters Using Contemporary Coexistence Theory.” Trends in Ecology & Evolution 2: 74–84. 10.1016/j.tree.2017.11.002 29180041

[ele70108-bib-0041] Goldberg, E. E. , and R. Lande . 2007. “Species' Borders and Dispersal Barriers.” American Naturalist 2: 297–304. 10.1086/518946 17874380

[ele70108-bib-0042] Gotelli, N. J. , and G. R. Graves . 1996. Null Models in Ecology. Smithsonian Institution Press.

[ele70108-bib-0135] Gotzenberger, L. , F. de Bello , K. A. Bråthen , et al. 2012. “Ecological Assembly Rules in Plant Communities – Approaches, Patterns, and Prospects.” Biological Reviews 87: 111–127. 21692965 10.1111/j.1469-185X.2011.00187.x

[ele70108-bib-0043] Gow, J. L. , C. L. Peichel , and E. B. Taylor . 2006. “Contrasting Hybridization Rates Between Sympatric Three‐Spined Sticklebacks Highlight the Fragility of Reproductive Barriers Between Evolutionarily Young Species.” Molecular Ecology 15: 739–752. 16499699 10.1111/j.1365-294X.2006.02825.x

[ele70108-bib-0044] Grainger, T. N. , A. D. Letten , B. Gilbert , and T. Fukami . 2019. “Applying Modern Coexistence Theory to Priority Effects.” Proceedings of the National Academy of Sciences of the United States of America 116: 6205–6210. 30850518 10.1073/pnas.1803122116 PMC6442631

[ele70108-bib-0045] Grant, P. R. 1972. “Divergent and Convergent Character Displacement.” Biological Journal of the Linnean Society 4: 39–68.

[ele70108-bib-0046] Grant, P. R. , and B. R. Grant . 2008. How and Why Species Multiply. Princeton University Press.

[ele70108-bib-0047] Greather, G. , and R. M. Grey . 1996. “Novel Cost of a Sexually Selected Trait in the Rubyspot Damselfly *Hetaerina americana*: Conspicuousness to Prey.” Behavioral Ecology 7: 465–473.

[ele70108-bib-0136] Grime, J. P. 2006. “Trait Convergence and Trait Divergence in Herbaceous Plant Communities: Mechanisms and Consequences.” Journal of Vegetation Science 17: 255–260.

[ele70108-bib-0048] Gröning, J. , and A. Hochkirch . 2008. “Reproductive Interference Between Animal Species.” Quarterly Review of Biology 83: 257–282. 18792662 10.1086/590510

[ele70108-bib-0049] Hardin, G. 1960. “The Competitive Exclusion Principle.” Science 131: 1292–1297. 14399717 10.1126/science.131.3409.1292

[ele70108-bib-0050] Hochkirch, A. , J. Gröning , and A. Bücker . 2007. “Sympatry With the Devil: Reproductive Interference Could Hamper Species Coexistence.” Journal of Animal Ecology 76: 633–642. 17584368 10.1111/j.1365-2656.2007.01241.x

[ele70108-bib-0051] Hollander, J. , M. Montaño‐Rendón , G. Bianco , et al. 2018. “Are Assortative Mating and Genital Divergence Driven by Reinforcement?” Evolution Letters 2: 557–566. 30564439 10.1002/evl3.85 PMC6292706

[ele70108-bib-0052] Holmes, R. T. , and F. A. Pitelka . 1968. “Food Overlap Among Coexisting Sandpipers on Northern Alaska Tundra.” Systematic Zoology 17: 305–318.

[ele70108-bib-0053] Holt, R. D. 2003. “On the Evolutionary Ecology of Species' Ranges.” Evolutionary Ecology Research 5: 159–178.

[ele70108-bib-0054] Hopkins, R. 2013. “Reinforcement in Plants.” New Phytologist 197: 1095–1103. 23495388 10.1111/nph.12119

[ele70108-bib-0055] Howard, D. J. 1993. “Reinforcement: Origin, Dynamics and Fate of an Evolutionary Hypothesis.” In Hybrid Zones and the Evolutionary Process, edited by R. G. Harrison . Oxford University Press.

[ele70108-bib-0056] Hubbell, S. P. 2001. The Unified Neutral Theory of Biodiversity and Biogeography. Princeton University Press. 10.1016/j.tree.2011.03.024 21561679

[ele70108-bib-0057] Irwin, D. 2020. “Assortative Mating in Hybrid Zones Is Remarkably Ineffective in Promoting Speciation.” American Naturalist 195: E150–E167. 10.1086/708529 32469663

[ele70108-bib-0058] Irwin, D. , and D. Schluter . 2022. “Hybridization and the Coexistence of Species.” American Naturalist 200: E93–E109. 10.1086/720365 35977784

[ele70108-bib-0059] Kearns, A. M. , M. Restani , I. Szabo , et al. 2018. “Genomic Evidence of Speciation Reversal in Ravens.” Nature Communications 9: 906. 10.1038/s41467-018-03294-w PMC5834606 29500409

[ele70108-bib-0060] Kottler, E. J. , E. E. Dickman , J. P. Sexton , N. C. Emery , and S. J. Franks . 2021. “Draining the Swamping Hypothesis: Little Evidence That Gene Flow Reduces Fitness at Range Edges.” Trends in Ecology & Evolution 36: 533–544. 33745756 10.1016/j.tree.2021.02.004

[ele70108-bib-0061] Kraft, N. J. B. , P. B. Adler , O. Godoy , E. C. James , S. Fuller , and J. M. Levine . 2014. “Community Assembly, Coexistence and the Environmental Filtering Metaphor.” Functional Ecology 29: 592–599.

[ele70108-bib-0063] Kyogoku, D. 2015. “Reproductive Interference: Ecological and Evolutionary Consequences of Interspecific Promiscuity.” Population Ecology 57: 253–260.

[ele70108-bib-0064] Lack, D. 1947. Darwin's Finches. Cambridge University Press.

[ele70108-bib-0065] Leibold, M. A. , M. Holyoak , N. Mouquet , et al. 2004. “The Metacommunity Concept: A Framework for Multi‐Scale Community Ecology.” Ecology Letters 7: 601–613.

[ele70108-bib-0066] Lewin, R. 1983. “Santa Rosalia Was a Goat.” Science 221: 636–639. 17787727 10.1126/science.221.4611.636

[ele70108-bib-0138] Linck, E. , B. G. Freeman , and J. B. Dumbacher . 2020. “Speciation and Gene Flow Across An Elevational Gradient in New Guinean Kingfishers.” Journal of Evolutionary Biology 33: 1643–1652. 32916016 10.1111/jeb.13698

[ele70108-bib-0067] Liou, L. W. , and T. D. Price . 1994. “Speciation by Reinforcement of Premating Isolation.” Evolution 48, no. 5: 1451–1459. 10.1111/j.1558-5646.1994.tb02187.x. 28568419

[ele70108-bib-0068] Losin, N. , J. P. Drury , K. S. Peiman , C. Storch , and G. F. Grether . 2016. “The Ecological and Evolutionary Stability of Interspecific Territoriality.” Ecology Letters 19: 260–267. 26757047 10.1111/ele.12561

[ele70108-bib-0069] Losos, J. B. 1990. “A Phylogenetic Analysis of Character Displacement in Caribbean Anolis Lizards.” Evolution 44: 558–569. 28567973 10.1111/j.1558-5646.1990.tb05938.x

[ele70108-bib-0070] Losos, J. B. 1992. “The Evolution of Convergent Structure in Caribbean Anolis Communities.” Systematic Biology 41: 403–420.

[ele70108-bib-0132] Losos, J. B. , S. Naeem , and R. K. Colwell . 1989. “Hutchinsonian Ratios and Statistical Power.” Evolution 43: 1820–1826. 28564336 10.1111/j.1558-5646.1989.tb02633.x

[ele70108-bib-0071] Louthan, A. M. , D. F. Doak , and A. L. Angert . 2015. “Where and When Do Species Interactions Set Range Limits?” Trends in Ecology & Evolution 30, no. 12: 780–792. 10.1016/j.tree.2015.09.011. 26525430

[ele70108-bib-0072] Macarthur, R. , and R. Levins . 1967. “The Limiting Similarity, Convergence, and Divergence of Coexisting Species.” American Naturalist 101: 377–385.

[ele70108-bib-0073] Mallet, J. 2005. “Hybridization as an Invasion of the Genome.” Trends in Ecology & Evolution 20: 229–237. 16701374 10.1016/j.tree.2005.02.010

[ele70108-bib-0074] Martin, P. R. , R. Montgomerie , and S. C. Lougheed . 2010. “Rapid Sympatry Explains Greater Color Pattern Divergence in High Latitude Birds.” Evolution 64: 336–347. 19744123 10.1111/j.1558-5646.2009.00831.x

[ele70108-bib-0075] Matute, D. M. , and B. S. Cooper . 2021. “Reinforcement Alone Does Not Explain Increased Reproductive Isolation in Sympatry.” bioRxiv. 10.1101/2021.05.06.442525.

[ele70108-bib-0076] Matute, D. R. , A. A. Comeault , E. Early , et al. 2020. “Rapid and Predictable Evolution of Admixed Populations Between Two *Drosophila* Species Pairs.” Genetics 214, no. 1: 211–230. 10.1534/genetics.119.302685. 31767631 PMC6944414

[ele70108-bib-0077] Matute, D. R. , and B. S. Cooper . 2020. “Comparative Studies on Speciation: 30 Years Since Coyne and Orr.” Evolution 75: 764–778. 10.1111/evo.14181 PMC8247902 33491225

[ele70108-bib-0078] Mayfield, M. M. , and J. M. Levine . 2010. “Opposing Effects of Competitive Exclusion on the Phylogenetic Structure of Communities.” Ecology Letters 13: 1085–1093. 20576030 10.1111/j.1461-0248.2010.01509.x

[ele70108-bib-0079] Mayr, E. 1947. “Ecological Factors in Speciation.” Evolution 1, no. 4: 263–288. 10.2307/2405327.

[ele70108-bib-0080] McEntee, J. P. , J. A. Tobias , C. Sheard , and J. G. Burleigh . 2018. “Tempo and Timing of Ecological Trait Divergence in Bird Speciation.” Nature Ecology & Evolution 2: 1120–1127. 29915344 10.1038/s41559-018-0570-y

[ele70108-bib-0081] Mikkelsen, E. K. , and D. Irwin . 2021. “Ongoing Production of Low‐Fitness Hybrids Limits Range Overlap Between Divergent Cryptic Species.” Molecular Ecology 30: 4090–4102. 34101940 10.1111/mec.16015

[ele70108-bib-0082] Milligan, B. G. 1985. “Evolutionary Divergence and Character Displacement in Two Phenotypically Variable Species.” Evolution 39: 1207–1222. 28564271 10.1111/j.1558-5646.1985.tb05687.x

[ele70108-bib-0083] Nishimura, T. , K. Terada , T. Xia , and Y. Takami . 2023. “Relationships Between Reproductive Character Displacement in Genital Morphology and the Population‐Level Cost of Interspecific Mating: Implications for the Templeton Effect.” Biological Journal of the Linnean Society 138: 14–26.

[ele70108-bib-0084] Noor, M. 1999. “Reinforcement and Other Consequences of Sympatry.” Heredity 83: 503–508. 10620021 10.1038/sj.hdy.6886320

[ele70108-bib-0085] Orr, H. 2009. “Fitness and Its Role in Evolutionary Genetics.” Nature Reviews. Genetics 10: 531–539. 10.1038/nrg2603 PMC2753274 19546856

[ele70108-bib-0086] Palumbi, S. R. 1994. “Genetic Divergence, Reproductive Isolation, and Marine Speciation.” Annual Review of Ecology and Systematics 25: 547–572.

[ele70108-bib-0087] Palumbi, S. R. 2008. “Speciation and the Evolution of Gamete Recognition Genes: Pattern and Process.” Heredity 102, no. 1: 66–76. 10.1038/hdy.2008.104. 19018273

[ele70108-bib-0091] Pfennig, D. W. , and R. A. Martin . 2010. “Evolution of Character Displacement in Spadefoot toads; Different Proximate Mechanisms in Different Species.” Evolution 64: 2331–2341. 20394671 10.1111/j.1558-5646.2010.01005.x

[ele70108-bib-0092] Pfennig, D. W. , and K. S. Pfennig . 2010. “Character Displacement and the Origins of Diversity.” American Naturalist 176: S26–S44. 10.1086/657056 PMC3285564 21043778

[ele70108-bib-0093] Pfennig, D. W. , and K. S. Pfennig . 2012. Evolution's Wedge: Competition and the Origins of Diversity. University of California Press.

[ele70108-bib-0094] Pigot, A. L. , and J. A. Tobias . 2013. “Species Interactions Constrain Geographic Range Expansion Over Evolutionary Time.” Ecology Letters 16: 330–338. 23231353 10.1111/ele.12043

[ele70108-bib-0095] Price, T. D. 2008. Speciation in Birds. Roberts.

[ele70108-bib-0097] Pulido‐Santacruz, P. , A. Aleixo , and J. T. Weir . 2018. “Morphologically Cryptic Amazonian Bird Species Pairs Exhibit Strong Postzygotic Reproductive Isolation.” Proceedings of the Royal Society B 285: 20172081. 29514967 10.1098/rspb.2017.2081 PMC5879619

[ele70108-bib-0140] Reijenga, B. R. , B. G. Freeman , D. J. Murrell , and A. L. Pigot . 2023. “Disentangling the Historical Routes to Community Assembly in the Global Epicentre of Biodiversity.” Global Ecology and Biogeography 23: 1748–1759.

[ele70108-bib-0099] Rensch, B. 1933. “Zoologische systematik und Artbildungsproblem.” Verhandlungen der Deutschen Zoologischen Gesellschaft, 19–83.

[ele70108-bib-0100] Rhymer, J. M. , and D. Simberloff . 1996. “Extinction by Hybridization and Introgression.” Annual Review of Ecology and Systematics 27: 83–109.

[ele70108-bib-0139] Rice, A. M. , and D. W. Pfennig . 2007. “Character Displacement: *In Situ* Evolution of Novel Phenotypes or Sorting of Pre‐Existing Variation?” Journal of Evolutionary Biology 20: 448–459. 17305810 10.1111/j.1420-9101.2006.01187.x

[ele70108-bib-0103] Runquist, R. B. , and M. L. Stanton . 2013. “Asymmetric and Frequency‐Dependent Pollinator‐Mediated Interactions May Influence Competitive Displacement in Two Vernal Pool Plants.” Ecology Letters 16: 183–190. 23134452 10.1111/ele.12026

[ele70108-bib-0104] Schalkowski, R. , K. Kasimatis , M. Greischar , and A. Cutter . 2025. “Reproductive Interference Alters Species Coexistence in Nematodes due to Asymmetric Sperm‐Induced Harm.” Ecology Letters 28, no. 1: e70067. 39901585 10.1111/ele.70067 PMC11791382

[ele70108-bib-0105] Schluter, D. 2000. The Ecology of Adaptive Radiation. Oxford University Press.

[ele70108-bib-0106] Schluter, D. , and J. D. McPhail . 1992. “Ecological Character Displacement and Speciation in Sticklebacks.” American Naturalist 140: 85–108. 10.1086/285404 19426066

[ele70108-bib-0107] Schumer, M. , R. Cui , D. L. Powell , G. G. Rosenthal , and P. Andolfatto . 2016. “Ancient Hybridization and Genomic Stabilization in a Swordtail Fish.” Molecular Ecology 25: 2661–2679. 26937625 10.1111/mec.13602

[ele70108-bib-0133] Seehausen, O. 2006. “Conservation: Losing Biodiversity by Reverse Speciation.” Current Biology 16: R334–R337. 16682344 10.1016/j.cub.2006.03.080

[ele70108-bib-0108] Servedio, M. R. , and M. A. F. Noor . 2003. “The Role of Reinforcement in Speciation: Theory and Data.” Annual Review of Ecology, Evolution, and Systematics 34, no. 1: 339–364. 10.1146/annurev.ecolsys.34.011802.132412.

[ele70108-bib-0109] Sexton, J. P. , P. J. McIntyre , A. L. Angert , and K. J. Rice . 2009. “Evolution and Ecology of Species Range Limits.” Annual Review of Ecology, Evolution, and Systematics 40: 415–436.

[ele70108-bib-0110] Slatkin, M. 1980. “Ecological Character Displacement.” Ecology 61: 163–177.

[ele70108-bib-0111] Smith, R. A. , and M. D. Rausher . 2008. “Experimental Evidence That Selection Favors Character Displacement in the Ivyleaf Morning Glory.” American Naturalist 171: 1–9. 10.1086/523948 18171146

[ele70108-bib-0112] Soni, S. P. , V. Apte , P. Joshi , and V. P. Cyriac . 2024. “Barking Up the Wrong Frog: Global Prevalence of Misdirected Amplexus in Anuran Amphibians.” Biological Journal of the Linnean Society: blae062.

[ele70108-bib-0113] Stone, L. , and A. Roberts . 1990. “The Checkerboard Score and Species Distributions.” Oecologia 85: 74–79. 28310957 10.1007/BF00317345

[ele70108-bib-0114] Strong, D. R. , L. A. Szyska , and D. S. Simberloff . 1979. “Tests of Community‐Wide Character Displacement Against Null Hypothesis.” Evolution 33: 897–913. 28568434 10.1111/j.1558-5646.1979.tb04743.x

[ele70108-bib-0115] Stroud, J. T. , S. T. Giery , R. J. P. Heathcote , et al. 2024. “Observing Character Displacement From Process to Pattern in a Novel Vertebrate Community.” Nature Communications 15: 9862. 10.1038/s41467-024-54302-1 PMC11564967 39543139

[ele70108-bib-0117] Stuart, Y. E. , S. A. Inkpen , R. Hopkins , and D. I. Bolnick . 2017. “Character Displacement Is a Pattern: So, What Causes It?” Biological Journal of the Linnean Society 121: 711–715.

[ele70108-bib-0118] Stuart, Y. E. , and J. B. Losos . 2013. “Ecological Character Displacement: Glass Half Full or Half Empty?” Trends in Ecology & Evolution 28: 402–408. 23537690 10.1016/j.tree.2013.02.014

[ele70108-bib-0119] Székely, A. J. , and S. Langenheder . 2014. “The Importance of Species Sorting Differs Between Habitat Generalists and Specialists in Bacterial Communities.” FEMS Microbiology Ecology 87: 102–112. 10.1111/1574-6941.12195. 23991811

[ele70108-bib-0120] Taylor, E. B. , J. W. Boughman , M. Groenenboom , M. Sniatynski , D. Schluter , and J. L. Gow . 2006. “Speciation in Reverse: Morphological and Genetic Evidence of the Collapse of a Three‐Spined Stickleback ( *Gasterosteus aculeatus* ) Species Pair.” Molecular Ecology 15: 343–355. 16448405 10.1111/j.1365-294X.2005.02794.x

[ele70108-bib-0121] Templeton, A. R. 1981. “Mechanisms of Speciation – A Population Genetic Approach.” Annual Review of Ecology and Systematics 12: 23–48.

[ele70108-bib-0122] Tobias, J. A. , C. K. Cornwallis , E. P. Derryberry , S. Claramunt , R. T. Brumfield , and N. Seddon . 2014. “Species Coexistence and the Dynamics of Phenotypic Evolution in Adaptive Radiation.” Nature 506: 359–363. 24362572 10.1038/nature12874

[ele70108-bib-0123] Todesco, M. , M. A. Pascual , G. L. Owens , et al. 2016. “Hybridization and Extinction.” Evolutionary Applications 9: 892–908. 27468307 10.1111/eva.12367 PMC4947151

[ele70108-bib-0124] Uyeda, J. C. , T. F. Hansen , S. J. Arnold , and J. Pienaar . 2011. “The Million Year Wait for Macroevolutionary Bursts.” Proceedings of the National Academy of Sciences of the United States of America 108: 15908–15913. 21873251 10.1073/pnas.1014503108 PMC3179053

[ele70108-bib-0125] Wallace, A. R. 1889. Darwinism: An Exposition of the Theory of Natural Selection; With Some of Its Applications. Macmillan and Co.

[ele70108-bib-0126] Webb, C. O. , D. D. Ackerly , M. A. McPeek , and M. J. Donoghue . 2002. “Phylogenies and Community Ecology.” Annual Review of Ecology and Systematics 33, no. 1: 475–505. 10.1146/annurev.ecolsys.33.010802.150448.

[ele70108-bib-0127] Weber, M. G. , and Y. S. Strauss . 2016. “Coexistence in Close Relatives.” Annual Review of Ecology, Evolution, and Systematics 47: 359–381.

[ele70108-bib-0128] Weiher, E. , and P. Keddy . 1995. “Assembly Rules, Null Models, and Trait Dispersion: New Questions From Old Patterns.” Oikos 74, no. 1: 159–164. 10.2307/3545686.

[ele70108-bib-0129] Weir, J. T. , and T. D. Price . 2011. “Limits to Speciation Inferred From Times to Secondary Sympatry and Ages of Hybridizing Species Along a Latitudinal Gradient.” American Naturalist 177: 462–469. 10.1086/658910 21460568

[ele70108-bib-0137] Wilson, J. B. 2007. “Trait‐Divergence Assembly Rules Have Been Demonstrated: Limiting Similarity Lives! A Reply to Grime.” Journal of Vegetation Science 18: 451–452.

[ele70108-bib-0130] Wu, W. , H. P. Lu , A. Sastri , et al. 2018. “Contrasting the Relative Importance of Species Sorting and Dispersal Limitation in Shaping Marine Bacterial Versus Protist Communities.” ISME Journal 12: 485–494. 29125596 10.1038/ismej.2017.183 PMC5776463

[ele70108-bib-0131] Yukilevich, R. 2012. “Asymmetrical Patterns of Speciation Uniquely Support Reinforcement in *Drosophila* .” Evolution 66: 1430–1446. 22519782 10.1111/j.1558-5646.2011.01534.x

